# Chronic Granulomatous Disease Presenting as Aseptic Ascites in a 2-Year-Old Child

**DOI:** 10.1155/2013/927897

**Published:** 2013-01-28

**Authors:** J. F. Moreau, John A. Ozolek, P. Ling Lin, Todd D. Green, Elaine A. Cassidy, Veena L. Venkat, Andrew R. Buchert

**Affiliations:** ^1^School of Medicine, University of Pittsburgh, Pittsburgh, PA 15261, USA; ^2^Division of Pathology, Children's Hospital of Pittsburgh of UPMC, One Children's Hospital Drive, 4401 Penn Avenue, Pittsburgh, PA 15224, USA; ^3^Division of Infectious Disease, Children's Hospital of Pittsburgh of UPMC, One Children's Hospital Drive, 4401 Penn Avenue, Pittsburgh, PA 15224, USA; ^4^Division of Pulmonary Medicine, Allergy and Immunology, Children's Hospital of Pittsburgh of UPMC, One Children's Hospital Drive, 4401 Penn Avenue, Pittsburgh, PA 15224, USA; ^5^Division of Rheumatology, Children's Hospital of Pittsburgh of UPMC, One Children's Hospital Drive, 4401 Penn Avenue, Pittsburgh, PA 15224, USA; ^6^Division of Gastroenterology, Children's Hospital of Pittsburgh of UPMC, One Children's Hospital Drive, 4401 Penn Avenue, Pittsburgh, PA 15224, USA; ^7^The Paul C. Gaffney Diagnostic Referral Service, Children's Hospital of Pittsburgh of UPMC, One Children's Hospital Drive, 4401 Penn Avenue, Pittsburgh, PA 15224, USA

## Abstract

Chronic granulomatous disease (CGD) is a rare inherited immunodeficiency syndrome that results from abnormal nicotinamide adenine dinucleotide phosphate (NADPH) oxidase function. This defect leads to recurrent catalase-positive bacterial and fungal infections as well as associated granuloma formation. We review the case of a 2-year-old boy who presented with ascites and fever of an unknown origin as manifestations of CGD. Cultures were negative for infection throughout his course, and CGD was suspected after identification of granulomas on peritoneal biopsy. Genetic testing revealed a novel mutation in the CYBB gene underlying his condition. This paper highlights the importance of considering CGD in the differential diagnosis of fever of unknown origin and ascites in children.

## 1. Introduction

Chronic granulomatous disease (CGD) results from the inability of neutrophils to complete the first step of the respiratory burst pathway, generation of superoxide, with the downstream consequence of impaired microbe killing. CGD leads to recurrent and potentially lethal infections. Pneumonia is the most common infection before diagnosis (47%) [1] and occurs in the majority (79%) of patients within four years of diagnosis [2]. Lymphadenitis is the second most common infection before diagnosis (45%) [1], and subcutaneous abscess is the second most common infection that occurs within 4 years of diagnosis (43%) [2]. Other presenting infections include osteomyelitis, liver and perirectal abscesses, enteritis, and septicemia [1]. CGD can, in very rare circumstances, present with ascites [3].

The incidence of CGD is estimated to be between 1/200,000 and 1/250,000 US births [2]. Most affected individuals are male because approximately 70% of mutations are X-linked recessive; the remaining 30% of cases are autosomal recessive [1, 2, 4]. In normal individuals, inflammatory stimuli prompt nicotinamide adenine dinucleotide phosphate (NADPH) oxidase (a lysosomal enzyme encoded on chromosome 22) to produce superoxide. Superoxide is converted to hydrogen peroxide via superoxide dismutase and then to hypochlorous acid (by myeloperoxidase), which is lethal to bacteria [5]. Individuals with CGD are able to utilize hydrogen peroxide made by microbes and convert it to hypochlorous acid to preserve microbe killing, yet catalase-positive bacteria can prevent this step by degrading the hydrogen peroxide. Thus, catalase-positive organisms such as *Staphylococcus aureus* are the most common microbial sources of infection; other common pathogenic organisms in patients with CGD are *Burkholderia cepacia*, *Nocardia*, *Serratia*, and *Klebsiella* [2, 6]. Approximately 70% of CGD cases are associated with mutations in the CYBB gene on the X chromosome, but well over 300 different CYBB mutations have been reported in association with the disease [7]. In this paper, we describe an unusual case of CGD in a child with a novel mutation in exon 13 of the CYBB gene who presented with an isolated lymphadenitis and months later developed ascites and culture-negative, granulomatous peritonitis.

## 2. Case Report

The patient is a 2-year-old male born to a G1P1 mother who had an uncomplicated pregnancy and delivery. His only significant past medical history was an isolated infection one and a half years earlier. His parents noticed a swollen area on his neck near his left ear and took him to our tertiary care children's hospital for evaluation. On admission, he was febrile to 39.3°C and had a leukocytosis of 20 × 10^9^/L. A diagnosis of lymphadenitis was made. A culture of the fluid drained from the abscess grew methicillin-sensitive *S. aureus,* but the blood culture was negative. He was discharged home on amoxicillin/clavulanate. The neck swelling returned after a few days despite oral antibiotics, prompting his parents to bring him back to the hospital. The abscess was drained for the second time, and he was administered intravenous ampicillin/sulbactam. With subsidence of the swelling, the patient was discharged home to complete a 10-day course of antibiotics and had no recurrence of the abscess.

In September 2011, he was admitted with nearly one month of daily fevers up to 39.4°C. His only other symptoms were intermittent dysuria and slightly loose stools at normal frequency (1-2 times daily). Family history of immunological, hematological, and oncological diseases was negative. There was no recent travel. Physical exam was within normal limits except for poor weight gain (decline from 36th to 7th percentile from 12 months of age to admission (28 months)). Abnormal laboratory values included albumin 2.8 g/dL (3.8–5.4 g/dL), alkaline phosphatase 509 IU/L (<290 IU/L), C-reactive protein (CRP) 10.69 mg/dL (0.08–1.2 mg/dL), erythrocyte sedimentation rate (ESR) 131 mm/hr (0–20 mm/hr), ferritin 178.1 ng/mL (10–60 ng/mL), hemoglobin (Hgb) 10.7 g/dL (11.5–13.5 g/dL), hematocrit 30.5% (34.0–40.0%), lactate dehydrogenase 258 IU/L (<171 IU/L), platelets 399 × 10^9^/L (156–369 × 10^9^/L), and neutrophils 57% (12–34%). All other laboratory values were within normal limits. The differential diagnoses included Kawasaki disease, hematological malignancy, early juvenile idiopathic arthritis or other rheumatologic process, and intestinal infection. Lyme disease, toxoplasmosis, Bartonella, cytomegalovirus, and tuberculosis were also considered. Subsequent testing excluded these infectious entities.

After a few days, he defervesced and was discharged home with presumptive diagnosis of a likely, yet to be diagnosed, rheumatological disease. One month later, he developed persistent fever and abdominal distension. Exam was now significant for increased abdominal girth, and a fluid wave was present on physical examination. Laboratory studies revealed an increased platelet count (457 × 10^9^/L) and ESR (74 mm/hr) as well as decreased ferritin (79.2 ng/mL) and Hgb (9.9 g/dL). His albumin also remained low, prompting testing for prealbumin (low at 5.6 mg/dL), proteinuria (negative), and protein losing enteropathy (alpha-1-antitrypsin normal (<20)). An abdominal CT scan showed contour irregularity of the colon, ascites, and diffuse peritoneal enhancement with fat stranding consistent with peritonitis. Paracentesis was done, and cytological evaluation demonstrated purulent fluid with large numbers of neutrophils and macrophages but no malignant cells. Bacterial and fungal cultures were sterile. The serum-to-ascites albumin gradient was 1 g/L, likely excluding portal hypertension as an etiology of the ascites. Gastrointestinal evaluation included esophagogastroduodenoscopy and colonoscopy with biopsies, endomysial antibody, amylase, and lipase. Duodenal, terminal ileal, and colonic biopsies were unremarkable and without villous abnormalities, inflammatory lymphoid cells, or accumulation of pigmented macrophages; the other studies were likewise normal. Bone marrow biopsy was unremarkable, and a tagged WBC scan demonstrated no specific abnormal foci of uptake. His abdomen remained distended, but the girth was stable. He defervesced on ampicillin/sulbactam and metronidazole and was discharged home to complete an empiric course of antibiotics for peritonitis.

**Figure 1:**
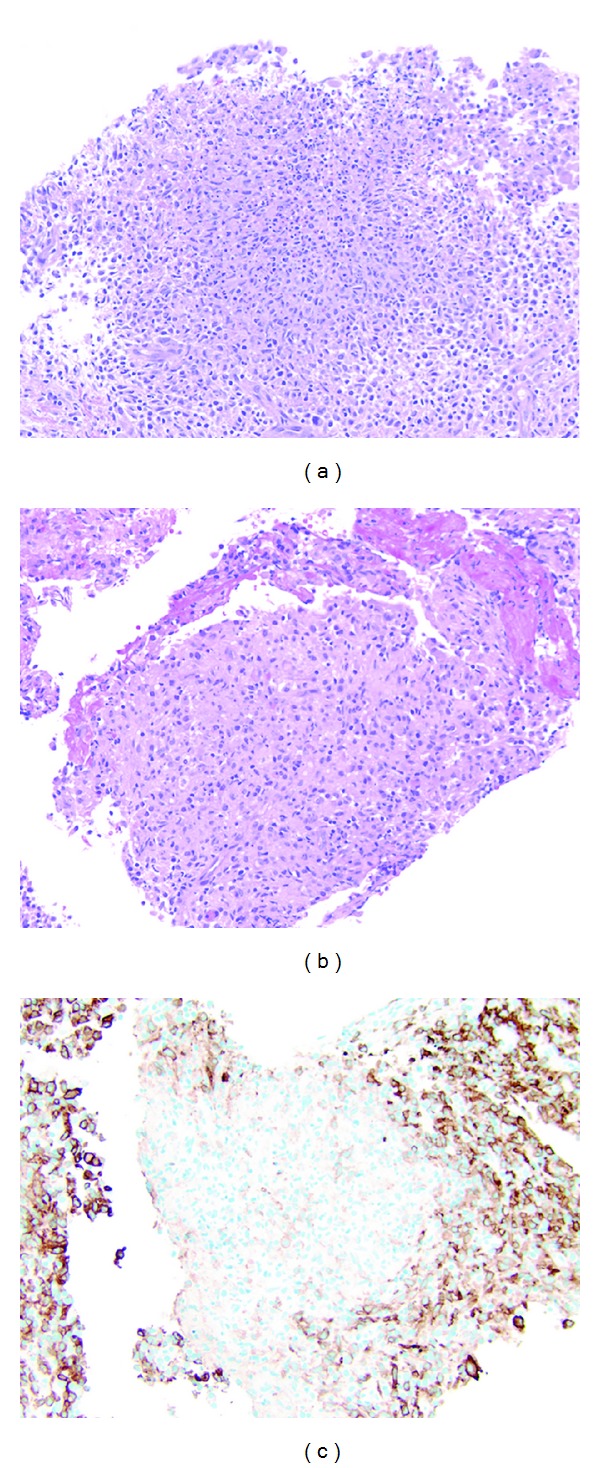
(a) Biopsy of peritoneal nodules demonstrated a cellular and organizing peritonitis including neutrophils, mesothelial cells, and abundant macrophages some with epithelioid features (HE, 200x). Focal areas had relatively well-organized granulomas (b) with macrophages that did not express the activated macrophage marker CD163 (c) ((b); HE, 200x, (c); CD163, 200x).

One week later, he again developed persistent fever and increasing abdominal distension and pain, resulting in his refusal to walk. An exploratory laparoscopy revealed massive ascites and an inflamed and friable peritoneum studded with yellow and white nodules. The ascitic fluid was sent for culture, and biopsies of the peritoneum were taken. The ascitic fluid had 1500 WBCs but was sterile. Pathology of the peritoneal biopsies revealed acute and organizing peritonitis with granulomatous features but no evidence of fungal, acid-fast, or bacterial microorganisms (Figure 1). The granulomatous nature of the peritonitis prompted evaluation for CGD. A dihydrorhodamine (DHR) test was performed to assess oxidase burst capability and resulted in negligible fluorescence, consistent with CGD. Genetic testing revealed hemizygosity for the c.1683dupG mutation in exon 13 of the CYBB gene.

## 3. Discussion

CGD was first described in 1957 independently by Berendes et al. [8] and by Landing and Shirkey [9] as a lethal disease in males associated with increased susceptibility to infection and pigment-containing macrophages in the visceral organs. The pigment forms as macrophages clear neutrophils that have undergone apoptosis, with subsequent cytoplasmic accumulation of ceroid pigment, hence their golden or orange-brown color by light microscopy [10]. In the 1960s and 1970s, defective NADPH oxidase was found to be the cause of CGD, leading to development of the nitroblue tetrazolium (NBT) test for diagnosis [11, 12]. A few decades later, the DHR was developed as more quantitative measure of oxidative burst, and it is now the preferred test for CGD [12]. Since then, over 400 mutations coding for the NADPH oxidase enzyme have been discovered, and genetic testing for CGD has become available.

Cases of CGD have been associated with defects in genes encoding 4 of the 6 NADPH oxidase subunits, named with reference to their molecular mass (kd) and “phox” for phagocyte oxidase. Flavocytochrome b558 is the redox center of NADPH oxidase and is composed of gp91phox and p22phox. Defects in the X-linked gene encoding gp-91 (CYBB) account for about 70% of known mutations causing CGD, and autosomal recessive p22phox mutations (CYBA gene on chromosome 16) account for an additional 5%. P47-phox (NCF1 gene on chromosome 7) and p67phox (NCF2 gene on chromosome 1) are both regulatory proteins that have also been associated with autosomal recessive defects (about 20% and 5%, resp.) [1, 2, 4, 13]. Rac2 (on chromosome 22) and p40phox (NCF4 gene on chromosome 22) are the other 2 NADPH oxidase subunits, and mutations have thus far not been associated with CGD.

We describe a child with CGD who presents with a rare constellation of fever of an unknown origin, ascites, and culture-negative peritonitis. To our knowledge, this is the first reported case of CGD associated with a c.1683dupG mutation in the CYBB gene [14, 15]. The c.1683dupG mutation in the CYBB gene causes a frameshift mutation that results in formation of a stop codon and thus loss of normal protein function. Ascites has rarely been reported as a presenting sign of chronic granulomatous disease, and, in all of the previously reported cases, bacterial cultures were positive [3, 16, 17]. Our patient did have nonspecific findings that are often present in patients with CGD (fever, microcytic anemia, failure to thrive). While he had a somewhat complicated lymphadenitis in the past, he lacked other infections and the chronicity typical of patients with CGD.

This paper highlights the importance of considering chronic granulomatous disease in the differential diagnosis of fever of unknown origin and ascites. With a broad differential diagnosis and little direct evidence of CGD before peritoneal biopsy, our patient reflects the challenge of making this diagnosis. The previously unreported mutation discovered in this patient may have been responsible for his unique clinical presentation. Past research has found that specific genetic polymorphisms are associated with development of gastrointestinal inflammation and rheumatologic disorders in CGD patients [18]. Other work has shown that the quantity of residual neutrophil reactive oxygen intermediates produced in CGD patients is mutation related and correlates with clinical outcomes [19]. In this context, knowing patient genotype may enable clinicians to provide improved care and is an area that we feel deserves further investigation.

## Abbreviations

CGD: Chronic granulomatous disease
NADPH: Nicotinamide adenine dinucleotide phosphate
CRP: C-reactive protein
ESR: Sedimentation rate
Hgb: Hemoglobin
WBC: White blood cell
DHR: Dihydrorhodamine
NBT: Nitroblue tetrazolium.
